# Minimally Invasive Direct Lateral Interbody Fusion (MIS-DLIF): Proof of Concept and Perioperative Results

**DOI:** 10.7759/cureus.979

**Published:** 2017-01-14

**Authors:** Hamid Abbasi, Ali Abbasi

**Affiliations:** 1 Tristate Brain and Spine Institute; 2 Trinity College, University of Cambridge

**Keywords:** interbody fusion, minimally invasive surgery, lumbar spine, operative surgical procedures, spine fusion

## Abstract

**Background:**

Minimally invasive direct lateral interbody fusion (MIS-DLIF) is a novel approach for fusions of the lumbar spine. In this proof of concept study, we describe the surgical technique and report our experience and the perioperative outcomes of the first nine patients who underwent this procedure.

**Study design/setting:**

In this study we establish the safety and efficacy of this approach. MIS-DLIF was performed on 15 spinal levels in nine patients who failed to respond to conservative therapy for the treatment of a re-herniated disk, spondylolisthesis, or other severe disk disease of the lumbar spine. We recorded surgery time, blood loss, fluoroscopy time, patient-reported pain, and complications.

**Methods:**

Throughout the MIS-DLIF procedure, the surgeon is aided by biplanar fluoroscopic imaging to place an interbody graft or cage into the disc space through the interpleural space. A discectomy is performed in the same minimally invasive fashion. The procedure is usually completed with posterior pedicle screw fixation.

**Results:**

MIS-DLIF took 44/85 minutes, on average, for 1/2 levels, with 54/112 ml of blood loss, and 0.3/1.7 days of hospital stay. Four of nine patients did not require overnight hospitalization and were discharged two to four hours after surgery. We did not encounter any clinically significant complications. At more than ninety days post surgery, the patients reported a statistically significant reduction of 4.5 points on a 10-point sliding pain scale.

**Conclusions:**

MIS-DLIF with pedicle screw fixation is a safe and clinically effective procedure for fusions of the lumbar spine. The procedure overcomes many of the limitations of the current minimally invasive approaches to the lumbar spine and is technically straightforward. MIS-DLIF has the potential to improve patient outcomes and reduce costs relative to the current standard of care and therefore warrants further investigation. We are currently expanding this study to a larger cohort and documenting long-term outcome data.

## Introduction

Chronic lower back pain affects about 60% of people in the United States during their lifetime [1] resulting in estimated economic costs of $635 billion [2]. Lumbar interbody fusion is a common surgical treatment for lower back pain caused by degenerative disk disease, disk herniation, and other pathologies of the spine. For lumbar interbody fusion, the spine can be approached from a variety of angles [3], but recently, extreme lateral interbody fusion (XLIF) [4-6], also known as direct lateral interbody fusion (DLIF), has gained popularity. In this approach, the patient is positioned in the lateral decubitus position and the disk is approached vertically, downwards, from an incision in the flank directly lateral to the disk.

XLIF procedures are usually accomplished using pedicle screw fixation and a variety of instrumentation, often necessitating repositioning the patient into the prone position [6]. Changing patient position alone increases the duration of the surgery by approximately 30–60 minutes, as it requires removal of the sterile draping for repositioning and subsequent redraping of the operative field. Therefore, the traditional direct lateral approach generally does not decrease surgical time and may in fact add to operative time. The XLIF procedure can be difficult to accomplish in L4-L5 because of the density of the lumbosacral plexus. The XLIF approach can be obstructed by the ribs for T12-L1 and L1-L2 based on patient habitus and is impractical and contraindicated in L5-S1 in the traditional fashion as the sacral ala and illiac crest block the approach [5]. These technical difficulties currently limit the applicability of XLIF. Furthermore, XLIF is a mini-open procedure requiring direct visualization. Open spinal surgeries cause higher surgical morbidity than minimally invasive (MIS) procedures, because the open approach disrupts connective tissue and muscle that stabilize the spine. Additionally, open surgeries require lengthier hospital stays and recovery times [7-8]. These factors increase the overall cost of open surgeries relative to their MIS equivalents [9-10].

In this study, we report on the feasibility and safety of a new MIS surgery for fusions of the lumbar spine called minimally invasive direct lateral interbody fusion (MIS-DLIF). Unlike traditional DLIF, MIS-DLIF does not require intraoperative repositioning of the patient and it can be performed safely and effectively in all levels between T12-S1. We establish the technique and safety of MIS-DLIF and report our experience with the first nine MIS-DLIF cases on a total of 15 spinal levels.

MIS-DLIF is similar to oblique lumbar lateral interbody fusion (OLLIF) [11]. Unlike OLLIF, the disk is not approached through Kambin’s triangle. Instead, the disk is approached from a more lateral angle, meaning the disk space is accessed anterior, rather than posterior, to the exiting nerve root. Therefore, MIS-DLIF can be used when Kambin’s triangle is too small for an OLLIF approach, either due to the patient’s innate anatomy or because of severe listhesis. Additionally, a true OLLIF approach is very difficult to the L5-S1 disk spaces because the ala of the sacrum and illiac crest block the approach, whereas the more lateral approach in MIS-DLIF is frequently possible. Thus, MIS-DLIF can be used as a rescue technique for OLLIF in L4-S1.

## Materials and methods

### Study design

This study includes nine MIS-DLIF patients to establish proof of concept (POC) and feasibility. Although MIS-DLIF has been utilized numerous times as backup for OLLIF, in this study, we only include patients in which we planned and executed the technique from start. The study was approved by the Pearl Institutional Review Board (Indianapolis, IN 46260) on 8/30/2016. Extensive consent was obtained from all patients. The surgeries were performed in Douglas County Hospital, Maple Grove Surgery Center, and Fairview Ridges Hospital.

### Patient selection

MIS-DLIF is indicated for patients with a re-herniated disk, spondylolisthesis, or severe disk disease of the lumbar spine, who have failed to respond to conservative therapy for four to six months and in whom discography has confirmed the origin of pain in the level of question. All patients have gone through a full course of conservative therapy before being considered as a surgical candidate. Exclusion criteria were significant vertebral anatomic abnormalities like scoliosis and hypokyphosis, which by themselves are not contraindications for DLIF, but we are reporting those patients in a different series.

Preoperative imaging included magnetic resonance imaging (MRI), X-ray of the thoracic spine with flexion and extension, a discogram, and a computerized axial tomography (CAT) scan. The patient demographics are listed in Table 1 and the number of fusions by level are shown in Table 2.

**Table 1:** MIS-DLIF patient demographics

# Cases	9
# Spinal Levels	15
# Male	6
# Smoker	2
Mean BMI	31
Mean Age	50

**Table 2:** MIS-DLIF surgery levels

Level	#
T12-L1	1
L1-L2	0
L2-L3	2
L3-L4	3
L4-L5	6
L5-S1	3

### Perioperative outcomes

Skin-to-skin surgery time, blood loss, and fluoroscopy times were recorded by clinic staff and entered into the electronic medical record (EMR) database immediately after surgery. Because no suction is used in MIS-DLIF, blood loss was measured by weighing sponges and subtracting dry weight.

### OR setup

**Figure 1:**
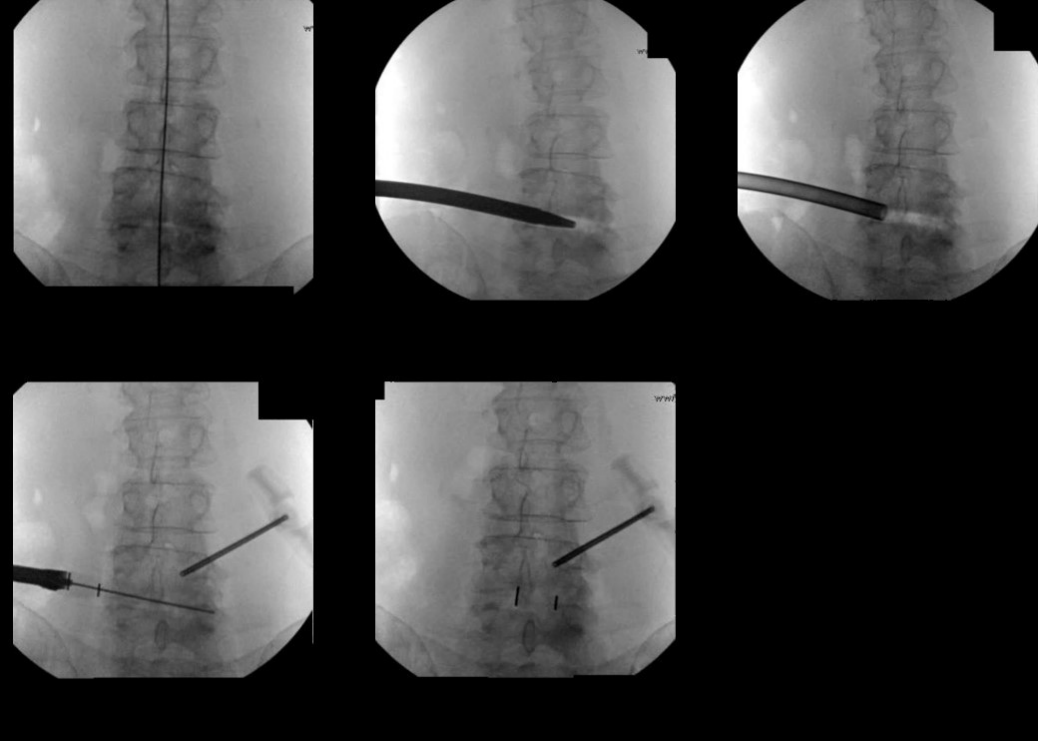
DLIF in the Fluoroscopic AP view

The patient is placed in the prone position on the operating table. To simplify the approach, the patient is tilted away from the surgeon by three to five degrees until the cage is inserted. For MIS pedicle screw fixation, the patient is planed into the true prone position. To enable quick readjustment, we used 3M Ioban (3M Center, MN, USA) transparent plastic draping which helps the surgeon obtain an appropriate understanding of the patient’s positioning and anatomy. Next, bilateral fluoroscopy is set up. The endplates of the target level should line up well in the lateral and be identifiable in the anterior-posterior (AP) view. Additionally, the spinous process should be centered between the pedicles in the AP view. The stages of a MIS-DLIF in the AP view are displayed in Figure 1. Electrophysiological monitoring is set up on the major muscle groups and skull. Somatosensory evoked potential (SSEP) is checked and monitored throughout the surgery.

### Marking

**Figure 2:**
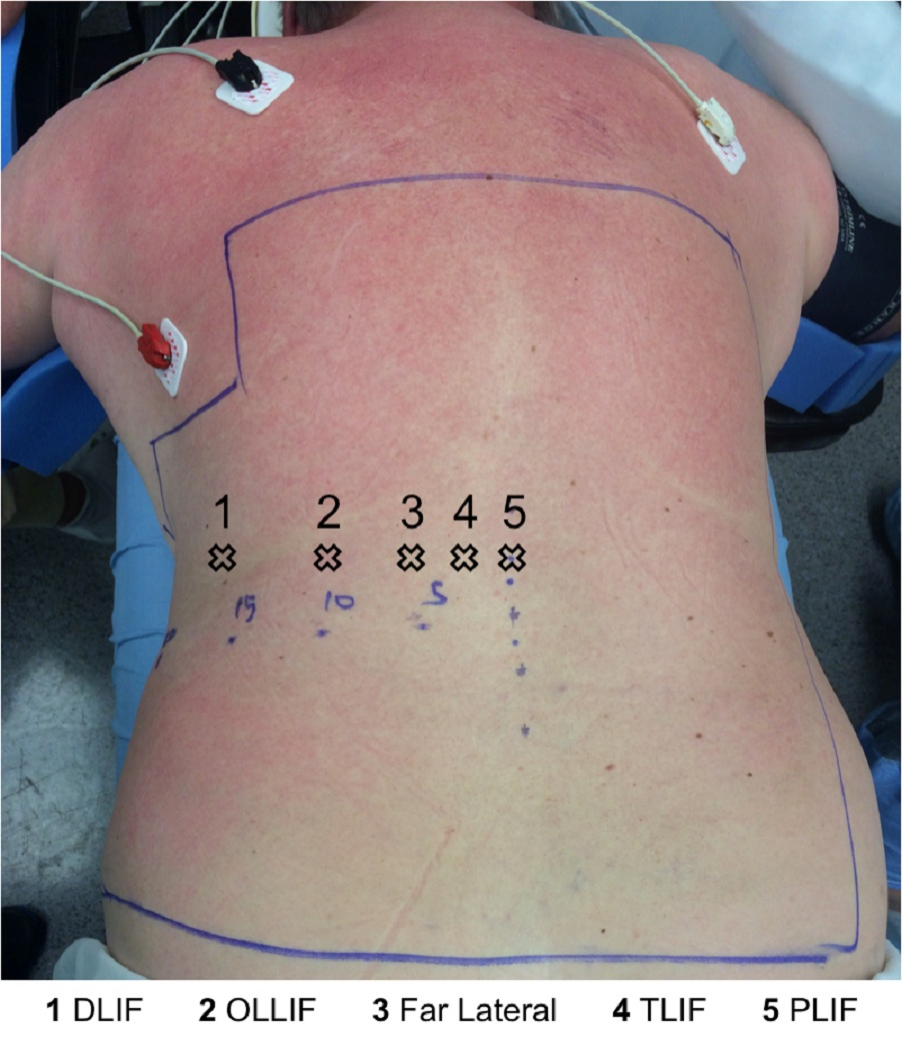
Trajectory points for different procedures: MIS-DLIF is the furthest on the left. Trajectory is usually perpendicular to the skin. Incision corresponds to above as well. Except for TLIF, both medial and paralateral (4 and 5) can, and has been, utilized.

In the AP view, the midline and all disks of interest are marked. Additionally, in the lateral view, a vertical line ending at the midpoint of each disk is marked. The midpoint of the disk (point A) and the posterior third of the disk (point B) are projected onto the skin in the lateral view. The incision is placed halfway between point A and point B. In most patients, the incision is 15–20 cm from the midline. The approximate incision point is shown in Figure 2. Multiple levels can be approached through the same incision by mobilizing the underlying skin and muscle and through the strategic placement of the incision.

### Approach

**Figure 3:**
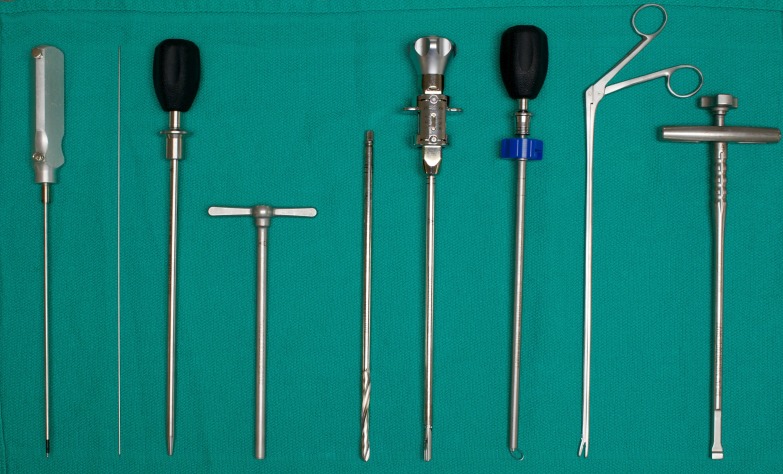
Instrumentation used for MIS-DLIF (left to right): Probe, K wire, dilator, working tube, disk drill, rotating curette, ring curette, rongeur, cage insertion device

**Figure 4:**
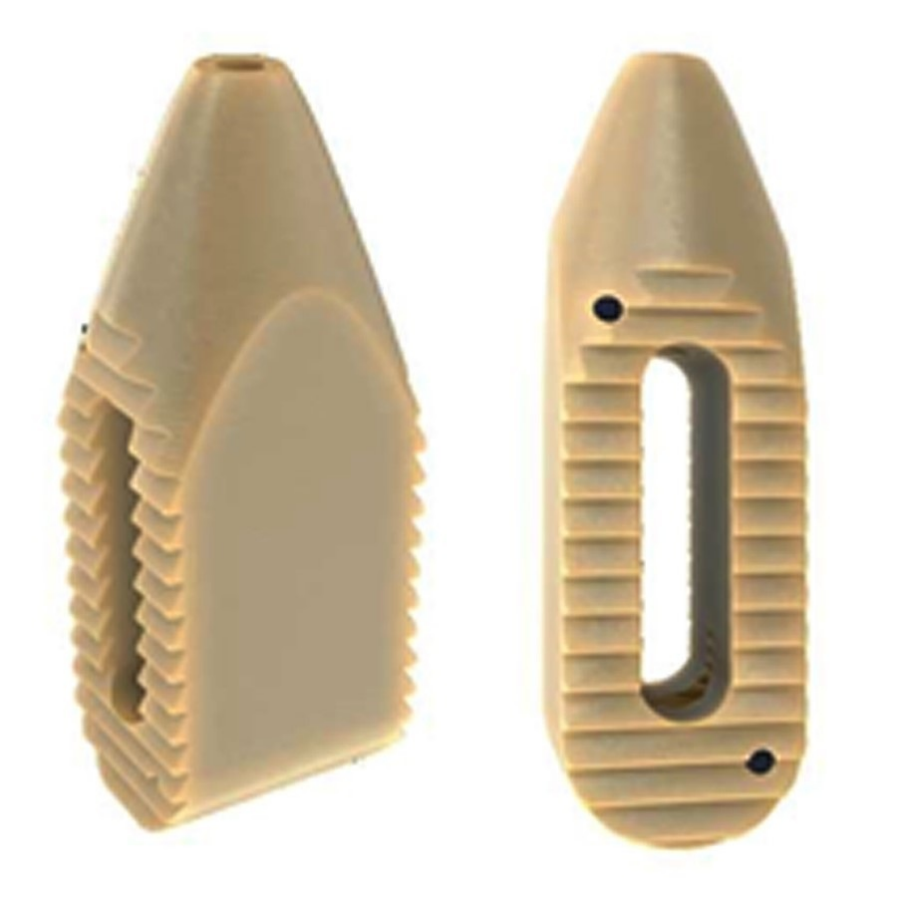
PEEK Zeus-O cage with bullet-nosed tip

**Figure 5:**
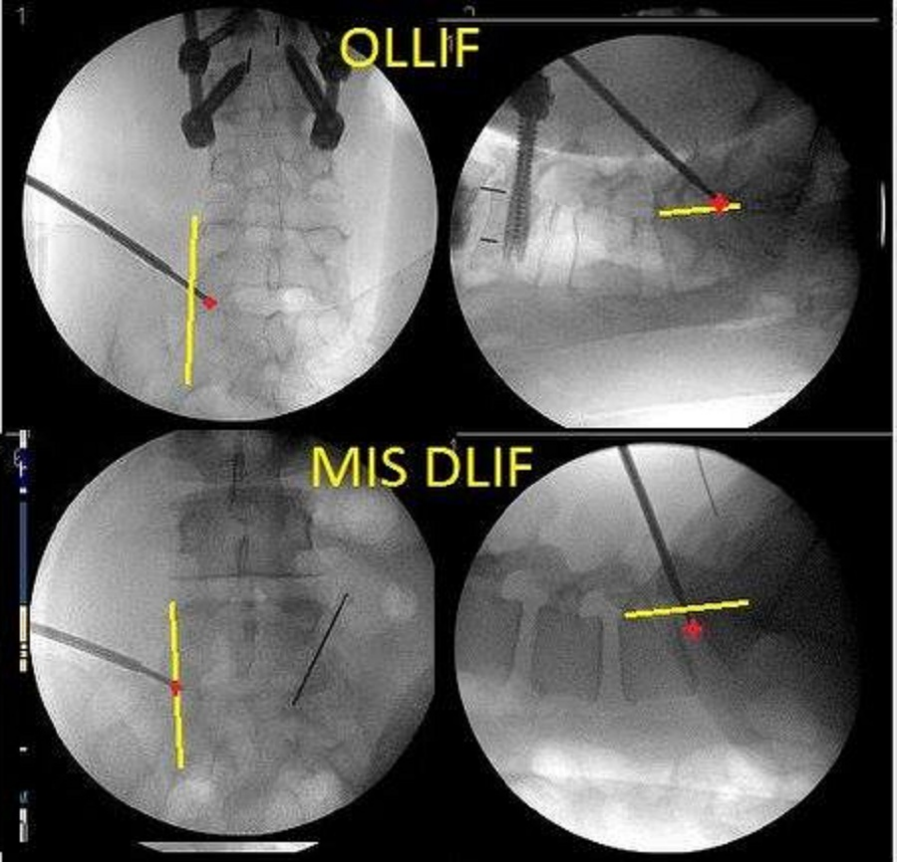
In OLLIF (top), the tip of the electrode is medial to border of the vertebral body (VB) in the AP view and right at the posterior aspect of the VB in the lateral view whereas in MIS-DLIF the tip of the electrode is at the lateral border of the VB in the AP view and anterior to the border of the VB in the lateral view

Instrumentation is shown in Figure 3. Aided by fluoroscopy, an electrode is entered through the incision and gently pushed through the underlying muscle and fascia. This blunt electrode traverses the retroperitoneal space until it is positioned on the iliopsoas muscle. The initial approach is similar, but quantifiably different, from OLLIF. In OLLIF, the tip of the electrode is medial to the border of the vertebral body (VB) in the AP view and exactly at the posterior aspect of the VB in lateral view. In MIS-DLIF, the tip of the electrode is exactly at the lateral border of the VB in the AP view, and anterior to the posterior border of the VB in the lateral view as shown in Figure 5. The surgeon verifies there is no contact with nerve structure in the lumbar plexus by stimulating at 3-4 mA and measuring nerve activities in the lower extremities [12]. Once a silent window is found, the electrode is advanced to the top of the disk and a sleeve is pushed down over the electrode. The electrode is removed and a K-wire is inserted into the disk past the midline. The sleeve is removed and a dilator is introduced over the K-wire. Under continued electrophysiological monitoring, the dilator is entered with careful circular motion, gently dilating the tissue, including the iliopsoas muscle. Next, a 10 mm working tube, called the access portal, is delivered over the dilator. The access portal is tapped into the disk until it passes the pedicle in the AP view. We used the Zeus-O instrumentation system (Amendia, Inc., GA, USA), as displayed in Figure 4, but any cannulated cage may be used.

### Discectomy and cage placement

The discectomy is performed entirely through the access portal. Disk material is removed first with a drill and then with a rotating cutter, ring curette, and long pituitary. The endplates are prepared with serial dilation of the rotating curette. Tactile feedback from the curette indicates when the endplates are reached, and it is important to ensure the endplates are free. Light violation of the endplates is desirable to aid in fusion and slight bleeding is not uncommon at this point. Next, the disk space is packed with tricalcium phosphate (TCP) (Berkeley Advanced Biomaterials Inc., CA, USA) soaked in autologous bone marrow aspirate, drawn from a Jamshidi needle in one of the pedicles.

A K-wire is placed once again and the access portal removed. At this stage, cage width and height can be determined using a trial spacer. However, with some experience, the cage dimensions can also be determined during discectomy through tactile feedback from the rotating cutter which has markings that indicate how wide the blades are spread.

Next, the cage (Figure 4, PEEK Zeus-O cage (Amendia, Inc., GA, USA)) is inserted over the K-wire aided by fluoroscopy (Figure 1). The conical shape of the cage ensures it passes through muscle and fascia and gently pushes the nerve root out of the way. With mallet taps, the cage is entered until 1/3 of the cage is past the midline. Some electrophysiological activity is not unusual during cage entry.

**Figure 6:**
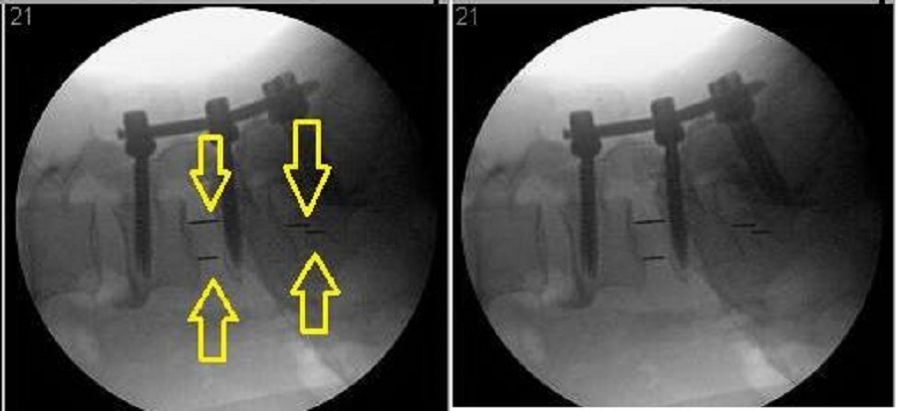
The cage profile in MIS-DLIF is more horizontal (L5/S1) compared to OLLIF (L4/5)

Compared to OLLIF, the final position of the cage is more horizontal as seen in Figure 6.

### Posterior pedicle screw fixation

After cage placement, all patients undergo percutaneous posterior pedicle screw fixation. We used Savannah-T posterior instruments and high top screws manufactured by Amendia (Amendia, Inc., GA, USA). The technique is similar to what has been described by Foley, et al. in 2001 [13], but we have modified it slightly to improve the odds of posterolateral fusion.

Jamshidi needles have already been placed in one level at the beginning of the surgery to allow tricalcium phosphate to be saturated with bone marrow aspirate. Once the cage is inserted, all pedicles are tapped with Jamshidi needles that are stimulated above 18 mA to assure there is no contact to neural structures. We ensure all Jamshidi needles are positioned correctly because repositioning is easiest at this point. We then place K-wires through the Jamshidi needles, and once the positioning of all K-wires is confirmed, we remove the AP fluoroscopic arm to ease screw placement.

Aided by lateral fluoroscopy, an osteotome with a groove is slid down the K-wire and the facets are bare boned. Next, a small amount of dry tricalcium phosphate is placed in the space just created on the facets. To complete the surgery, the screws are inserted and the rod is placed as described by Foley, et al. [13].

## Results

### Perioperative outcomes

Full perioperative results are shown in Table 2. MIS-DLIF takes 44/85 minutes, on average, for 1/2 levels, with 54/112 ml of blood loss, and 0.3/1.7 days of hospital stay. A total of four out of the nine patients did not require overnight hospitalization and were discharged two to four hours after surgery in very good condition.

### Complications

No significant complications were encountered during surgery. Two patients reported nerve irritation post surgery that improved upon follow-up. One patient reported slowly improving nerve irritation three months post surgery and was subsequently lost to follow-up.

### Patient reported pain

Data is shown in Table 3. Before surgery, the patients reported average pain of 9.3 ± 0.9 on a 10 point scale. At the most recent follow-up (between 90–349 days post surgery) the pain was reduced significantly to 4.8 ± 2.3 (one sided, paired t-test p<0.001). On the Oswestry Disability Index (ODI) [14], patients improved from 62 ± 16% pre-surgery to 32 ± 20% post surgery (one-sided, paired t-test p<0.001).

**Table 3:** MIS-DLIF perioperative outcomes

	# of Levels	
	1	2
# Cases	3	6
OP Time (min)	44 ± 4	85 ± 19
Bloodless (ml)	54 ± 28	112 ± 101
Fluoroscopy (s)	186 ± 34	472 ± 212
Hospital Stay (days)	0.3 ± 0.6	1.7 ± 1.4

## Discussion

In the initial patient group, MIS-DLIF has been a safe and clinically effective procedure. From a surgeon’s perspective, MIS-DLIF is easier to master than comparative MIS fusions, because it does not require direct visualization, fascetectomy, or laminectomy.

Table 4 compares the perioperative outcomes in MIS-DLIF to two systematic reviews of minimally invasive fusion surgeries [15-16] totaling 30 different studies. The surgery time and hospital stay we report for MIS-DLIF are significantly lower than those reported in any of the reviewed studies. The blood loss for MIS-DLIF is at the lower end of the reviewed studies. Given that, even with very conservative monitoring, four out of nine patients did not require overnight hospitalization, MIS-DLIF may soon be routinely performed as an outpatient procedure.

**Table 4:** Comparison of perioperative outcomes in MIS-DLIF with literature reviews of MIS-TLIF and MIS-PLIF

	MIS-DLIF 1/2 level	Goldstein 2014 [16] range	Karikari 2010 [15] range (n=7)
OP Time (min)	44/85	104-390 (n=23)	156-348
Bloodless (ml)	54/112	51-496 (n=23)	150-456
Hospital Stay (days)	0.3/1.7	1.8-11 (n=21)	3-10.6

This study includes only nine cases, which is not sufficient to demonstrate the long-term efficacy of this procedure. However, it should be noted that except for a different approach angle, MIS-DLIF is identical to OLLIF, a procedure that this surgeon has performed over 400 times on more than 700 total spinal levels. Like OLLIF, MIS-DLIF allows the surgeon to pack the disk space with TCP or a biologic because the procedure only creates a small opening in the disk space that is lateral to the nerve root and completely sealed by the cage. We hypothesize this leads to higher fusion rates and, together with the MIS nature of the procedure, to lower rates of adjacent level disease and reoperation. We are currently collecting long-term outcome data on OLLIF to test this hypothesis.

OLLIF has already been shown to be less expensive than open surgeries due to reduced OR time and hospital stays [10]. We hypothesize that the same savings will apply to MIS-DLIF, especially given the short hospital stay described in this study. We are currently collecting data on the long-term results and economics of MIS-DLIF and will publish those findings in a subsequent study.

This is a proof of concept study for a novel approach to fusion of the lumbar spine. Although MIS-DLIF is similar to a previously published procedure (OLLIF), but there are enough differences in the approach and applicability of MIS-DLIF to warrant separate study of the long-term efficacy of MIS-DLIF. We are aware that our initial sample size is small and are expanding the study cohort and collecting long-term outcome data, such as disability indices and fusion rates.

## Conclusions

MIS-DLIF is a minimally invasive equivalent to DLIF/XLIF fusion of the lumbar spine. In MIS-DLIF, the disk space is approached laterally, aided by biplanar fluoroscopy and electrophysiological monitoring. The procedure is complemented with MIS pedicle screw fixation. Unlike previous MIS fusions of the lumbar spine, this procedure can safely be performed on T12/L1, L4-L5 and L5-S1. Unlike in DLIF/XLIF, repositioning and redraping of the patient is not necessary and this saves significant time during the surgery. This is a proof of concept study on 15 spinal levels with nine patients. We have found that MIS-DLIF is safe, effective, and extremely fast, taking on average only 44 minutes for a single level, and 85 minutes for two level procedures. No patient stayed in the hospital for more than three days, and four patients did not require an overnight stay. MIS-DLIF is a promising procedure with the potential for reduced rates of complications and reoperation and significant cost savings. MIS-DLIF warrants further study with a larger cohort and long-term follow-up.
